# Nanozyme-armored natural enzymes for acute kidney injury management via inflammation regulation and oxidative damage mitigation

**DOI:** 10.3389/fphar.2025.1686323

**Published:** 2025-12-18

**Authors:** Juntao Wang, Ruifeng Li, Xiaofei Fu, Shuai Huo, Lijiao Wang, Fengmin Shao, Yue Gu

**Affiliations:** 1 Department of Nephrology, The First People’s Hospital of Shangqiu, Shangqiu, China; 2 Academy of Medical Sciences, Tianjian Laboratory of Advanced Biomedical Sciences, Zhengzhou University, Zhengzhou, China; 3 Department of Nephrology, Henan Clinical Medical Research Center for Nephropathy, Henan Provincial Key Laboratory of Kidney Disease and Immunology, Henan Provincial People’s Hospital, Zhengzhou, China; 4 Department of Nephrology, Central China Subcenter of National Center for Cardiovascular Diseases, Henan Cardiovascular Disease Center, Fuwai Central‐China Cardiovascular Hospital, Central China Fuwai Hospital of Zhengzhou University, Zhengzhou, China

**Keywords:** acute kidney injury, inflammation, nanozyme, reactive oxygen species, sulfuretted hydrogen

## Abstract

**Background:**

Acute kidney injury (AKI) is a major global disease with a complex pathogenesis and a lack of safe and effective radical treatments. Reactive oxygen species (ROS) and inflammation are key drivers of AKI, making antioxidant and anti_inflammatory strategies promising. This study designed a stable nanozyme to simultaneously scavenge ROS and control inflammation.

**Methods:**

Using a biomimetic mineralization approach, catalase (CAT) was immobilized on manganese_based metal sulfide to construct the CAT@MnS nanozyme. *In vitro* experiments were conducted to assess its antioxidant activity and protective effect on HEK293 cells. *In vivo*, cisplatin_ and rhabdomyolysis_induced AKI mouse models were used to evaluate renal targeting, ROS_scavenging capacity, and anti_inflammatory efficacy.

**Results:**

*In vitro*, CAT@MnS effectively scavenged ROS and protected HEK293 cells from oxidative stress. *In vivo*, it accumulated in kidneys, reduced renal ROS, lowered inflammatory cytokine levels, and alleviated kidney damage in both AKI models.

**Discussion:**

This study successfully developed a CAT@MnS nanozyme that integrates ROS scavenging with inflammation regulation. The biomimetic mineralization strategy enhanced the stability and therapeutic synergy of the system. The nanozyme offers a new approach for the synergistic treatment of AKI and shows promising potential for translational application.

## 1 Introduction

Acute kidney injury (AKI) is an acute inflammatory disease of the kidney, which is characterized by sudden (within 1–7 days) or persistent (>24 h) rapid decline in renal function, with high morbidity and mortality (Kellum et al., 2021a; Kellum et al., 2021b; Thadhani et al., 1996). In recent years, the incidence of AKI has been on the rise, it affects up to 15.3% of all hospitalized patients (Waikar et al., 2006). In the intensive care setting, 1-year mortality was five times higher among patients with acute AKI that did not recover, compared to those with a history of rapidly reversible AKI. in non-critical care settings, 12-month mortality affected 64.9% of patients who developed AKI (Ozrazgat-Baslanti et al., 2021). AKI is one of the most serious diseases in the world due to its complex pathogenesis and the lack of safe and effective radical treatment strategies (Susantitaphong et al., 2013). AKI can be caused by trauma, sepsis, surgery, rhabdomyolysis syndrome or nephrotoxic drugs, among which nephrotoxic substances are one of the main causes (Kellum et al., 2021a; Su et al., 2025). As one of the largest metabolic organs in the human body, kidney is the main organ involved in the action of various toxic substances. When the cumulative amount or single dose of toxic substances reaches a certain level, the redox balance in the kidney will be destroyed, resulting in oxidative stress and obvious nephrotoxicity. This oxidative stress state further leads to the activation of apoptosis pathway, ultimately leading to cell death and AKI. In addition, the continuous progress of AKI can cause renal fibrosis, thus aggravate chronic kidney disease (CKD) and end-stage renal failure (ESRD) risk (Chawla and Kimmel, 2012; Xu et al., 2019). At present, the clinical interventions for AKI patients are mainly Renal Replacement Therapy (RRT) and nutritional supplementation. Although many agents such as diuretics, vasoactive agents, and growth factors have been reported in animal models or preliminary clinical studies, no effective treatment has been established. Therefore, there is an urgent need to seek new drugs to treat AKI.

According to studies, ROS plays a crucial role in the occurrence and development of AKI (Su et al., 2023). Under the stimulus environment of inflammation, kidney can produce a large number of ROS. ROS is an important class of chemical substances, which are formed from the incomplete reduction reaction of oxygen and are an important part of aerobic metabolism in the body (D'Autreaux and Toledano, 2007). ROS is composed of superoxide anion (O_2_^−^), hydrogen peroxide (H_2_O_2_), singlet oxygen, and hydroxyl radical (Nosaka and Nosaka, 2017; Yang et al., 2019). ROS mainly arise from the electron transport chain in the inner mitochondrial membrane during oxidative phosphorylation. Electron leakage at NADH dehydrogenase and III partially reduces oxygen, forming O_2_^−^. O_2_^−^ reacts with nitric oxide (NO) within mitochondria to produce peroxynitrite (ONOO^−^). To balance intracellular ROS levels, superoxide dismutases (SODs) catalyze O_2_^−^ disproportionation into hydrogen peroxide (H_2_O_2_), which is then degraded by enzymes like glutathione (GSH). Excess mitochondrial ROS can trigger the opening of the mitochondrial permeability transition pore, leading to the release of proteins such as cytochrome c from the inner mitochondrial membrane into the cytoplasm (Zorov et al., 2014). High Mobility Group Box 1 (HMGB1) is a highly conserved non-histone chromatin-associated protein that functions as a damage-associated molecular pattern (DAMP) molecule by binding to pattern recognition receptors, ROS-induced oxidative stress is one of the primary pathophysiological drivers of HMGB1-related functional disorders leading to the release of pro-inflammatory cytokines such as TNF-α, IL-6, and IL-1β (Andersson et al., 2018; Datta et al., 2024a; Datta et al., 2024b). Normal levels of intracellular ROS are important for maintaining intercellular signaling and intracellular physiological functions. However, under the imbalance of oxidative stress, renal ROS production exceeds the renal clearance capacity, which causes ultimately induces inflammation and apoptosis of tubular epithelial cells, and then disturbs the water-electrolyte balance and acid-base homeostasis of the body (Liu et al., 2020; Zhang et al., 2021). Therefore, antioxidant treatment is expected to become one of the effective targets of AKI.

There are enzyme systems and antioxidants to remove reactive oxygen species in the human body, such as superoxide dismutase (SOD), catalase (CAT), glutathione peroxidase (GPx), vitamin E, vitamin C, among which SOD, CAT, GPx are also known as natural enzymes (Hao et al., 2019). Natural enzymes can convert ROS into harmless products such as oxygen (O_2_) and water (H_2_O) (Ferreira et al., 2018). However, natural enzymes are easily inactivated, degraded, and have short half-life, which greatly limits their clinical application (Ratnam et al., 2006; Ramalho et al., 2020). In addition, most natural enzymes have difficulty in crossing the glomerular filtration barrier (GFB), further limiting their application in the treatment of AKI.

In recent years, with the development of nanomedicine, more and more attention has been paid to the study of using encapsulants such as liposomes, micelles or polymers to improve the stability of proteins (Lawrence and Price, 2016). In addition, the use of inorganic nanoparticles as delivery carriers can not only improve the retention time of proteins in the circulatory system, but also prevent the immune response caused by exogenous proteins.

In addition to eliminating excess ROS in the lesion site, inducing inflammatory cell apoptosis and inhibiting the expression of inflammatory factors are also crucial to reduce inflammatory injury caused by ROS. H_2_S is an endogenous gas signaling molecule that plays an important role in physiological and pathological processes. It has been widely used in the treatment of tumors, inflammation, central nervous system (CNS) diseases and other diseases. Increasing evidences show that H_2_S can induce apoptosis of neutrophils and inhibit the expression of some leukocytes and endothelial adhesion factors by activating KATP channels, thereby inhibiting the production of endogenous H_2_S and inducing leukocyte adhesion (Zanardo et al., 2006; Li et al., 2008). At the same time, H_2_S can regulate inflammatory responses by controlling the production of interleukin (IL)-10, IL-8 and IL-6 (Li et al., 2008). Therefore, designing and constructing new artificial antioxidants that can effectively enter the site of kidney injury and exert synergistic effects of antioxidant and anti-inflammatory is a potential strategy for the treatment of AKI.

**SCHEME 1:**
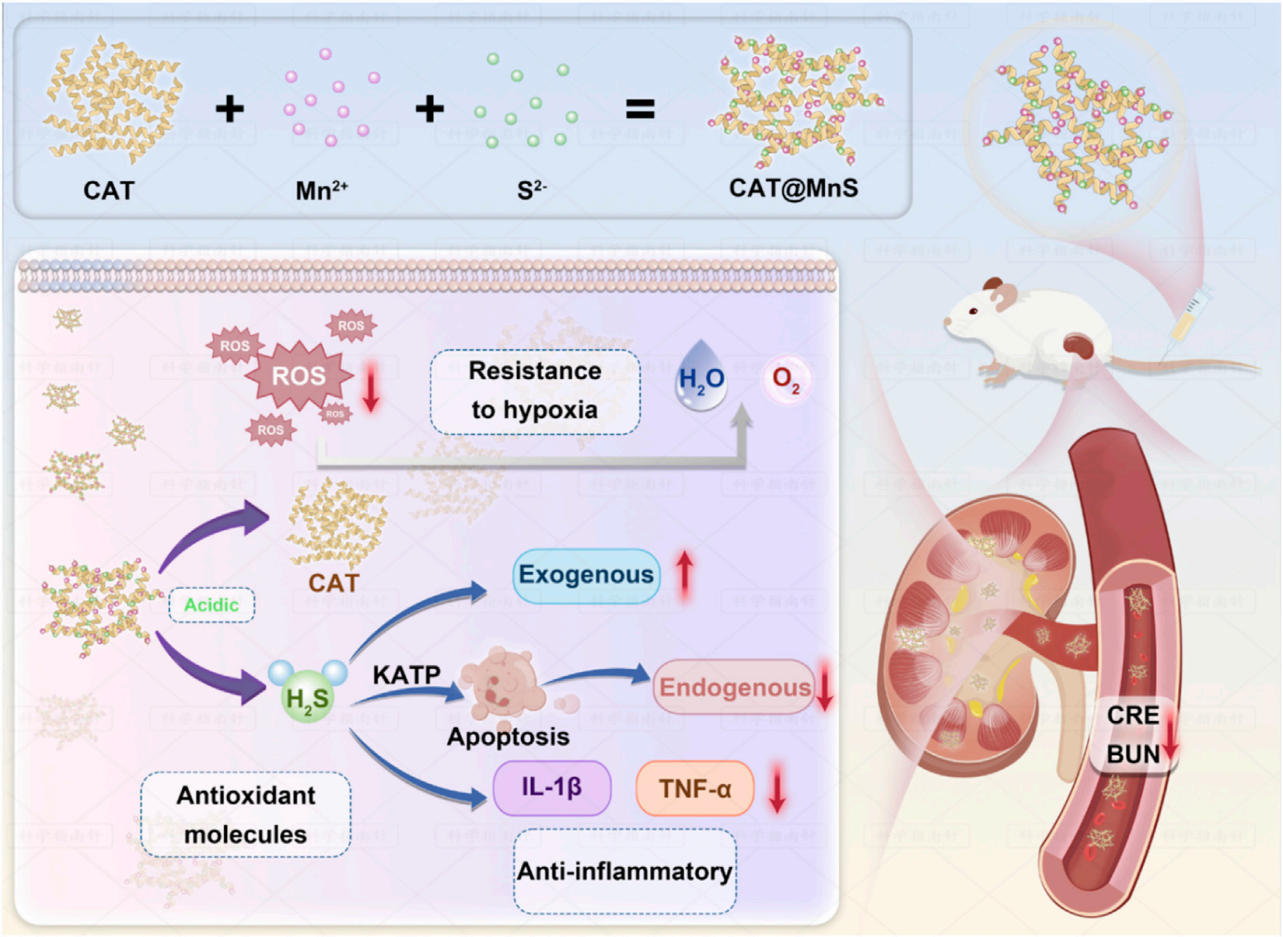
Schematic diagram of the synthesis of CAT@MnS and its mechanism of alleviating AKI through antioxidant and anti-inflammatory effects.

Manganese (Mn) as an important part of the metal nanozyme, due to its excellent biocompatibility and degradation in organisms, is considered to be the ideal candidate for the biological applications (Li et al., 2024; Singh et al., 2017; Chu et al., 2024). Here, we constructed a biodegradable manganese sulfide (MnS) immobilized catalase (CAT) nanocomposite named CAT@MnS by a biomimetic mineralization strategy. The nanocomposite can be decomposed under acidic conditions, releasing H_2_S and CAT. CAT@MnS can effectively protect AKI mice through GFB by antioxidant and anti-inflammatory effects (Scheme 1).

## 2 Materials and methods

### 2.1 Chemicals and materials

Bovine serum albumin (BSA), Catalase (CAT), Sodium sulfide (Na_2_S), Iron chloride hexahydrate (FeCl_3_·6H_2_O), Cupric chloride dihydrate (CuCl_2_·2H_2_O), Nickel chloride hexahydrate (NiCl_2_·6H_2_O), Zinc chloride (ZnCl_2_), Manganese Chloride Tetrahydrate (MnCl_2_·4H_2_O) were purchased from Shanghai Macklin Biochemical Technology Co., Ltd (China). Hydrogen peroxide (H_2_O_2_, 30%), ABTS, and DCFD-A were bought from Sigma-Aldrich (United States). Reactive Oxygen Species Assay Kit, Apoptosis Detection kit (propidium iodide/annexin V-FITC) were obtained from the Beyotime Institute of Biotechnology (China). All the aqueous solutions were prepared using purified deionized (DI) water purified with a purification system (Direct-Q3, Millipore, United States).

### 2.2 Synthesis of BSA@MnS

Briefly, 10 mg bovine serum albumin (BSA), 7.3 mg manganese chloride (MnCl_2_), and 8.9 mg sodium sulfide (Na_2_S) were measured. MnCl_2_ and Na_2_S were dissolved in 1 mL deionized water by magnetic stirring until completely dissolved and then placed for later use. BSA was dissolved in 1.2 mL deionized water and placed on a vortex oscillator to oscillate until the BSA solution was completely dissolved. Add 0.1 mL MnCl_2_ solution in shangbu solution, violent mixing after 15 min, slowly add 0.15 mL of Na2S solution; After the mixture solution was placed at 4 °C for 3 h, the solution turned black. The black solution was dialyzed in deionized water for 24 h using a dialysis bag with a truncated molecular weight of 8,000–14000, and then cooled and dried in a lyophilizing machine to obtain BSA@MnS nanomaterials.

### 2.3 Synthesis of CAT@MnS

Natural catalase (CAT) 10 mg, manganese chloride (MnCl_2_) 7.3 mg, and sodium sulfide (Na_2_S) 8.9 mg extracted from bovine liver were weighed. MnCl_2_ and Na_2_S were dissolved in 1 mL deionized water by magnetic stirring until completely dissolved and then placed for later use. The CAT enzyme was dissolved in 1.2 mL deionized water and shaken on a vortex oscillator until the CAT enzyme solution was completely dispersed. After centrifugation at 8,000 rpm for 5 min, the supernatant was taken for further use. 0.1 mL MnCl_2_ solution was added to the supernatant of the previous step, and after vigorous stirring for 15 min, 0.15 mL Na_2_S solution was slowly added. After the mixture solution was placed at 4 °C for 3 h, the solution turned black. After 24 h of dialysis using a dialysis bag with a molecular weight cutoff of 8,000–14000 in deionized water, the resulting material was dried in a lyophilizer to obtain CAT@MnS nanomaterials.

### 2.4 Detection of catalase-like activity

The analyte were dissolved in deionized water to prepare 20 μg/mL solutions. The reaction was initiated by the sequential addition of 10 mL of PBS, 200 μL of the analyte, followed by 200 μL of 0.3% H_2_O_2_. Oxygen generation was monitored at 1-min intervals over a period of 10 min. The oxygen generation curve was plotted with time as the X-axis and the oxygen generation (the difference in oxygen concentration at all time points from 0 min) as the Y-axis. The catalase activity was determined using a dissolved oxygen meter. The initial rate of oxygen production was recorded. One unit of catalase activity was defined as the amount of enzyme that produces 0.5 μmol of oxygen per minute under the assay conditions. The specific activity was expressed as units per milligram of protein (U/mg).

### 2.5 ABTS radical-scavenging activity of CAT@MnS and BSA@MnS

Mix the ABTS aqueous solution with potassium persulfate (K_2_S_2_O8) solution to prepare a mixed liquor concentration of 2.45 mM. Incubate in the dark at 4 °C for 16 h. The mixture is then combined with anhydrous ethanol. When the absorbance reaches 0.7 ± 0.02, it becomes the ABTS working solution. Mix 10 μL of Nano-enzyme at different concentrations (0, 0.0625, 0.125, 0.25, 0.5, 1, 2, 4 mg/mL) with 190 μL of the ABTS working solution, incubate at room temperature for 6 min, and then measure the absorbance at 735 nm.

### 2.6 Detection of H_2_S gas release

First, a standard curve for the release of H_2_S gas was constructed by preparing solutions of different concentrations using an appropriate amount of Na_2_S crystals dissolved in deionized water. The content of H_2_S gas in these solutions was then measured using a hydrogen sulfide (H_2_S) content detection kit and a UV-visible spectrophotometer to determine the absorbance (OD value). The X-axis represented the H_2_S gas content, while the Y-axis represented the OD values measured by the UV-visible spectrophotometer. The CAT@MnS nanomaterial was dispersed in a buffer solution with a pH of 6.5 at a concentration of 1 mg/mL and gently agitated at 37 °C. Subsequently, the H_2_S content in 2 mL of CAT@MnS solution was monitored at time intervals of 0.5, 1, 2, 3, 4, 5, 6, and 8 h. The corresponding OD values were determined using the H_2_S content detection kit within the UV-visible spectrophotometer and were used to calculate the corresponding H_2_S gas content based on the previously established standard curve for H_2_S gas release.

### 2.7 Hemolysis assay of CAT@MnS and BSA@MnS

Prepare Nano-enzyme into six solutions with concentrations of 50, 100, 200, 300, 400, and 600 μg/mL. Mix 900 μL of Nano-enzyme solutions at different concentrations with 100 μL of fresh red blood cell suspension, and incubate at 37 °C for 30 min. Set up a positive control (low ionic water, which will cause complete hemolysis of red blood cells) and a negative control (PBS, in which red blood cells maintain their normal morphology). After the incubation, remove the unhemolysed red blood cells by centrifugation, and take the supernatant to measure its absorbance at 578 nm. By calculating the hemolysis rate, the hemolytic ability of the nanomaterial can be quantitatively evaluated.

### 2.8 Cell culture

Human embryonic kidney 293 (HEK293) cells were purchased from the American Type Culture Collection (ATCC) and cultured under 5% CO_2_ at 37 °C in Dulbecco’s Modified Eagle Medium (DMEM) supplemented with 1% penicillin/streptomycin and 10% fetal bovine serum (FBS).

### 2.9 CCK-8 cell proliferation and cytotoxicity assay

The CCK-8 assay was employed to evaluate the protective effects of BSA@MnS, CAT enzyme, and CAT@MnS on HEK293 cell proliferation under conditions of hydrogen peroxide (H_2_O_2_)-induced damage.

Three experimental groups were established: (1) BSA@MnS + H_2_O_2_; (2) CAT + H_2_O_2_; (3) CAT@MnS + H_2_O_2_. Following digestion and centrifugation, HEK293 cells were resuspended in a complete culture medium at a concentration of 8 × 10^4^ cells/mL and plated at 100 μL per well in a 96-well plate using a complete culture medium. After plating, the cells were incubated for 24 h. The original drugs BSA@MnS, CAT enzyme, and CAT@MnS were diluted to seven concentration gradients of 5, 10, 20 and 40 μg/mL using a complete culture medium. Subsequently removing the original culture medium from each well was followed by sequential addition of drug solutions at each gradient with a volume of 50 μL. The plate was then placed in a cell culture incubator for 30 min after which 50 μL of 800 μM H_2_O_2_ solution diluted in complete culture medium was added to each well before further incubation for 12 h. The drug was applied at final concentrations of 2.5, 5, 10, and 20 μg/mL, while the final concentration of H_2_O_2_ was maintained at 400 μM. CCK-8 reagent was added post-incubation and OD values were measured using a Microplate reader.

### 2.10 AKI mouse model

The Balb/c female mice (6–8 weeks, 17–21 g) used in this study were purchased from the Experimental Animal Center of Zhengzhou University, and all animal studies were conducted by the protocol approved by the Animal Ethics Committee of the Experimental Animal Center of Zhengzhou University (No. ZZU-LAC20220729 [04]). All experiments were performed in accordance with relevant named guidelines and regulations. All authors complied with the ARRIVE guidelines.

Glycerol-induced AKI mouse model: Balb/c mice were dehydrated for 15 h but were able to freely obtain food. Afterward, 50% glycerol was injected into the muscles of both hind limbs of the mice at a dose of 8 mL/kg. After 2 h of injection, an AKI mouse model was successfully established and used for subsequent experiments.

Cisplatin-Induced AKI mouse model: cisplatin (20 mg/kg) injected into the abdominal cavity of all Balb/c mice. For the treatment group, mice were injected with BSA@MnS, CAT, and CAT@MnS through the tail vein 2 h after intraperitoneal injection of cisplatin.

After 24 h, each group of mice was euthanized by CO_2_ asphyxiation (50% flow rate of chamber volume per minute for CO_2_), and blood samples and kidney tissue were collected and analyzed.

### 2.11 Therapeutic effect in AKI mice

To analyze the therapeutic effects of BSA@MnS, CAT, and CAT@MnS, we randomly divided mice into five groups: (i) healthy mice treated with PBS; (ii) AKI mice treated with PBS; iii) AKI mice treated with CAT; (iv) AKI mice treated with BSA@MnS; (v) AKI mice treated with CAT@MnS. After treatment, kidney function and body weight were monitored.

### 2.12 Kidney function evaluation

After 24 h of intravenous injection of BSA@MnS, CAT, and CAT@MnS, the mice were euthanized using CO_2_ asphyxiation with 50% flow rate of chamber volume per minute for CO_2_, and kidney and blood samples were collected. The obtained kidney sections were stained with H&E, and the levels of BUN and CRE in blood samples were detected.

### 2.13 Evaluation of ROS clearance capacity

To further assess the antioxidant capacity of CAT@MnS at a microscale level, we investigated the mechanism of action by examining the clearance of ROS in the kidneys. Initially, the kidneys from each experimental group of mice were extracted and stored at −80 °C. Subsequently, cryosectioning was performed at 20 °C to obtain kidney tissue sections with a thickness of approximately 5 μm. The frozen sections were washed with PBS and stained with DHE fluorescent probe for 30 min to detect superoxide anion formation. Following this, coverslips were mounted on each slide using Vectashield Mounting Medium (Vector Laboratories, Burlingame, CA, United States), and confocal imaging was conducted using a laser scanning confocal microscope.

### 2.14 Study of drug distribution in the body

The normal mice were randomly divided into two groups (n = 3): (1) CAT; and (2) CAT@MnS. Cy5.5-labeled natural CAT and CAT@MnS were injected via the tail vein at a drug-to-body weight ratio in both groups. The mice in group 1 received injections of natural CAT, while those in group 2 received injections of CAT@MnS. Live imaging was performed on the experimental mice at 5 min, 0.5 h, 1 h, 3 h, 6 h, 8 h, and 24 h post-administration using an *in vivo* imaging system.

### 2.15 Ethics approval obtained

All animal studies were conducted by the protocol approved by the Animal Ethics Committee of the Experimental Animal Center of Zhengzhou University (No. ZZU-LAC20220729 [04]). All experiments were performed in accordance with relevant named guidelines and regulations. All authors complied with the ARRIVE guidelines.

### 2.16 Statistical analysis

Quantitative data were analyzed using GraphPad Prism software. All data are presented as mean ± standard error of the mean (SEM). Significance testing was performed using either Student’s unpaired t-test or one-way ANOVA followed by a post hoc test, and P < 0.05 was considered statistically significant.

## 3 Results and discussion

### 3.1 Construction and characterization of CAT@MnS nanoparticles

**FIGURE 1:**
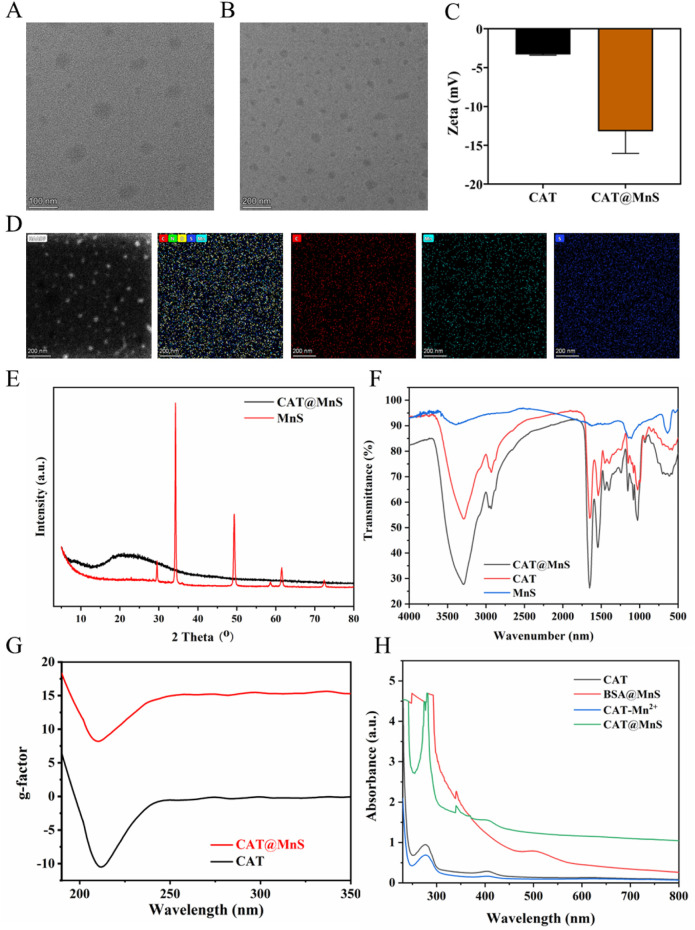
Structural characterization of CAT@MnS. **(A)** and **(B)** TEM images of CAT@MnS. **(C)** DLS results of CAT and CAT@MnS. **(D)** Elemental mapping of CAT@MnS. **(E)** PXRD image of CAT@MnS and MnS. **(F)** FTIR spectra of CAT@MnS, CAT and MnS. **(G)** CD images of CAT@MnS and CAT. **(H)** UV-visible of CAT, BSA@MnS, CAT-Mn_2_^+^ and CAT@MnS.

CAT@MnS and BSA@MnS are synthesized by a simple and economical method. The Transmission electron microscopy (TEM) images (Figures 1A,B) revealed that CAT@MnS consisted of uniformly spherical nanoparticles with an average particle size of 70 nm. After MnS packaging, the dynamic light scattering (DLS) results (Figure 1C) show that the Zeta potential of CAT decreases from −3.3 mV to −13.2 mV, confirming that we have successfully used the biomimetic mineralization method to fix MnS on the surface of CAT. Elemental mapping of CAT@MnS demonstrated the presence of Mn, and S signals in the complex (Figure 1D), this provides direct evidence that CAT loads MnS. The Powder X-Ray Diffractometer (PXRD) image (Figure 1E) showed that CAT@MnS was amorphous and had a different structural morphology from MnS. The Fourier Transform Infrared (FTIR) showed that new peaks appeared in CAT@MnS compared with separate CAT and MnS, further proving the successful insertion of MnS (Figure 1F). The circular dichroism (CD) image shows that the peak values of CAT@MnS and natural CAT are similar, which indicates that the CAT structure in CAT@MnS remains unchanged (Figure 1G). In addition, according to the UV-visible spectrum (UV-visible) shown in Figure 1H, a distinct absorption peak at approximately 280 nm can be observed for both CAT@MnS and CAT, indicating that CAT has successfully bound to CAT@MnS.

### 3.2 Antioxidative activities of CAT@MnS nanoparticles

**FIGURE 2:**
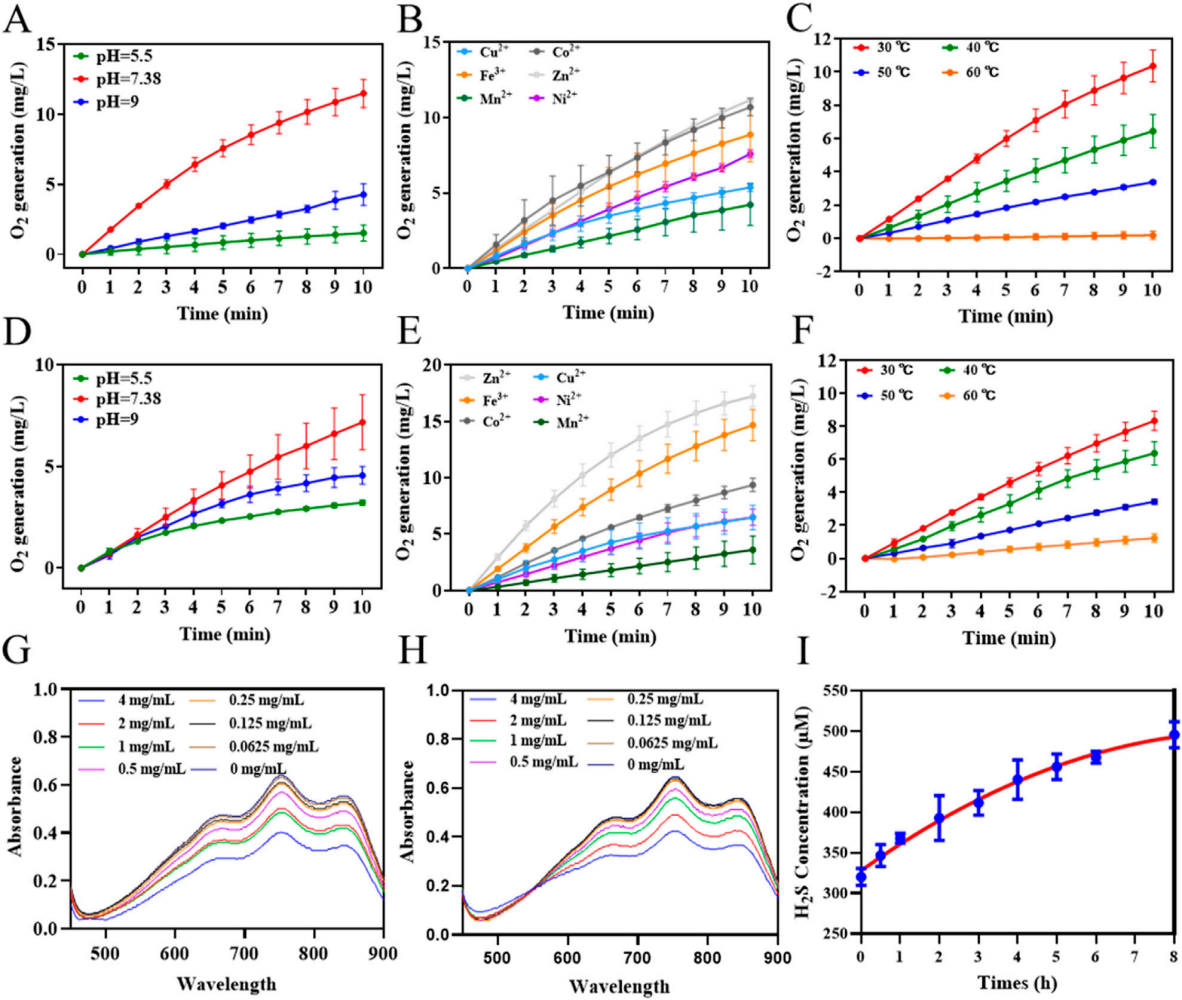
Antioxidative activities of CAT@MnS. **(A–C)** Influence of different pH, metal ion concentrations, and temperatures on CAT-like activity of CAT, n = 3. **(D–F)** Influence of different pH, metal ion concentrations, and temperatures on CAT-like activity of CAT@MnS, n = 3. **(G)** ABTS scanning spectra of CAT@MnS at different concentrations (mg/mL). The concentrations are 4, 2, 1, 0.5, 0.25, 0.125 and 0.0625 mg/mL, n = 3. **(H)** ABTS scanning spectra of BSA@MnS at different concentrations (mg/mL). The concentrations are 4, 2, 1, 0.5, 0.25, 0.125, and 0.0625 mg/mL. **(I)** H_2_S release line diagram of CAT@MnS.

To verify the stability of the CAT@MnS nanomaterial CAT-like activity. First, we measured the catalase-like activity of CAT@MnS and BSA@MnS using a dissolved oxygen meter. The results indicate that the catalase activity of CAT@MnS is significantly higher than that of BSA@MnS (Supplementary Figure S1). The oxygen generation assay revealed that CAT@MnS produced a denser array of bubbles than BSA@MnS, indicating its significantly higher catalase-like activity (Supplementary Figure S2; Supplementary Table S1). Then, we measured the CAT-like activity of CAT and CAT@MnS under different pH, metal ion concentrations, and temperatures. The results indicate that, compared to natural CAT, the change in the CAT-like activity of CAT@MnS under different pH, and temperatures is minimal (Figures 2A–F; Supplementary Figure S3). This proves that the CAT@MnS nanomaterial synthesized by biomimetic mineralization method has high stability of CAT-like activity.

ABTS via oxidation can generate a stable structure of blue-green ABTS free radicals, the maximum absorption peak of the ABTS free radical is located at 735 nm. When antioxidants were added to the reaction system, ABTS free radicals could react with them and fade the solution, and the absorption peak at 735 nm decreased. The decrease of the absorption peak was directly proportional to the degree of free radical scavenging. Therefore, this method can be used to detect the antioxidant capacity of various hydrophilic or hydrophobic substances. The ABTS assay results for CAT@MnS and BSA@MnS indicated that the MnS component imparts ABTS radical scavenging activity to both nanomaterials (Figures 2G,H; Supplementary Figure S4). H_2_S gas can regulate the expression of inflammatory factors and cells to produce anti-inflammatory effects. We used the reaction of H_2_S with N, N-dimethyl-p-phenylenediamine, and ammonium ferric sulfate to generate methylene blue to detect the change in the content of released H_2_S gas in the CAT@MnS solution over time. The results showed that as time went on, the content of H_2_S gas gradually increased and showed a sustained upward trend (Figure 2I).

### 3.3 Biological safety evaluation of CAT@MnS

In the evaluation of the biocompatibility of CAT@MnS, the hemolysis test is a crucial component. The hemolysis test aims to investigate whether CAT@MnS and BSA@MnS can cause red blood cell lysis, leading to the release of hemoglobin, thereby assessing its potential risks. Fresh mouse blood was used in the experiment, and the red blood cell suspension was prepared after anticoagulant treatment. Then, the red blood cell suspension was mixed and incubated with solutions of CAT@MnS and BSA@MnS at different concentrations, respectively. Positive (deionized water) and negative (PBS) control groups were also set up. After incubation at 37 °C for 30 min, the supernatant was collected by centrifugation, and the absorbance was measured at 735 nm using a microplate reader to calculate the hemolysis rate. The results showed that compared with the positive control group, the hemolysis rate of all CAT@MnS and BSA@MnS concentration groups was significantly lower than that of the positive group (Supplementary Figure S5). Even at the highest concentration, the hemolysis rate of CAT@MnS and BSA@MnS was still far below the safety threshold of 5%. The hemolysis rate of the negative control group was almost zero. These findings indicate that under the experimental conditions, CAT@MnS and BSA@MnS have minimal impact on the integrity of red blood cell membranes and do not cause significant hemolysis. From the perspective of the hemolysis test, this preliminarily demonstrates that CAT@MnS has good biocompatibility.

**FIGURE 3:**
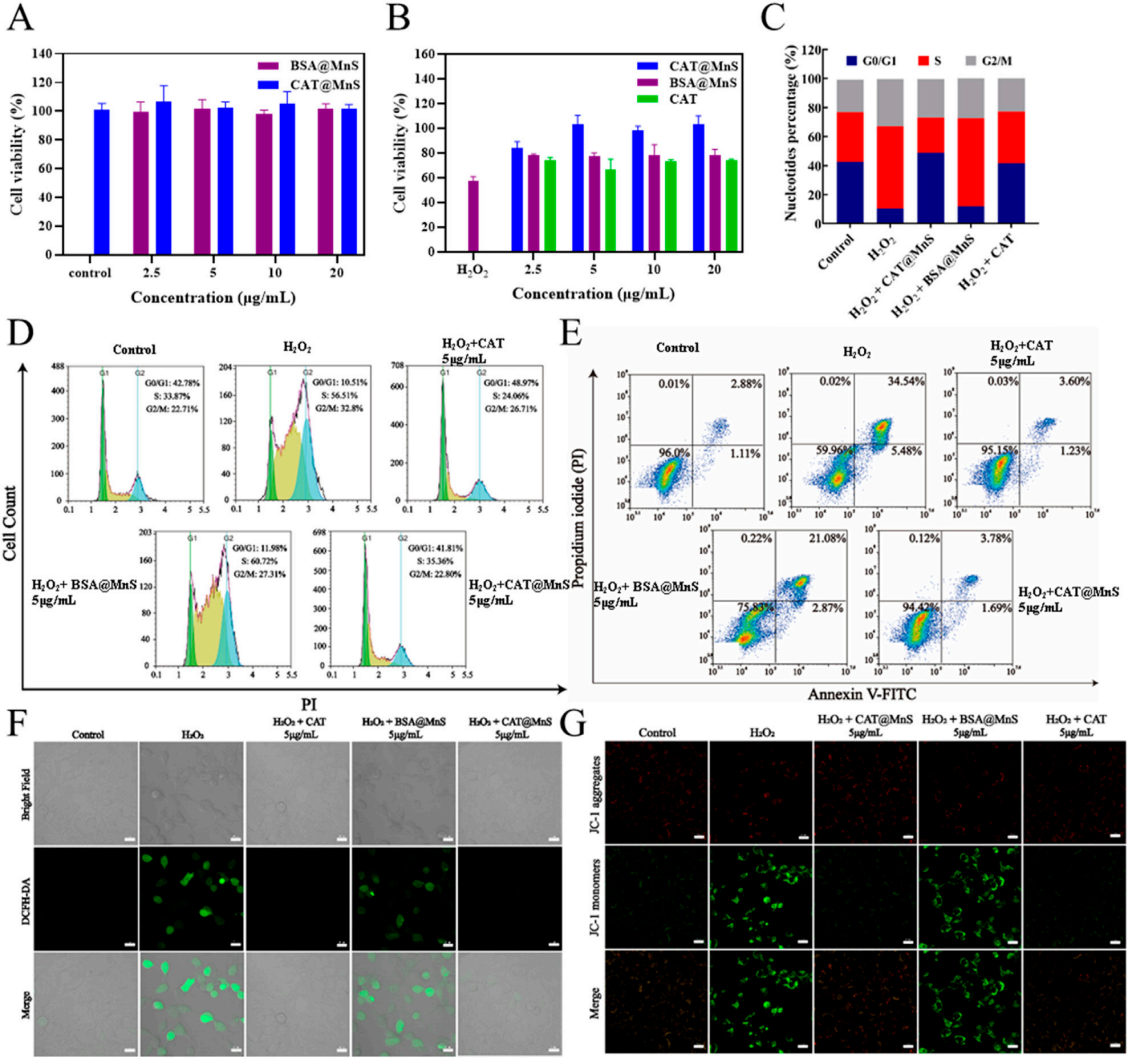
Antioxidant effects of CAT@MnS. **(A)** Drug toxicity gradient verification of CAT@MnS, n = 3. **(B)** Effectiveness gradient verification of CAT@MnS protection effect, H_2_O_2_ was used at 400 μM. n = 3. **(C**, **D)** Cell cycle and apoptosis analysis after treatment with different groups, n = 3. **(E)** Flow cytometry plot of cell apoptosis after treatment with different groups, n = 3. **(F)** Fluorescence images of ROS staining after treatment with different groups, n = 3. **(G)** Fluorescence images of MMP after treatment with different groups.

The safety of nanomaterials to cells is crucial. We used a CCK8 assay kit to detect the effects of CAT@MnS and BSA@MnS on the proliferation of HEK293 cells. As shown in Figure 3A, there was no significant decrease in the viability of HEK293 cells as the drug concentration increased, indicating that CAT@MnS and BSA@MnS have no obvious cytotoxicity to normal cells.

Furthermore, to verify the *in vitro* antioxidant therapeutic effects of CAT@MnS, we evaluated the protective effects of CAT@MnS, CAT, and BSA@MnS on the proliferation inhibition of HEK293 cells in H_2_O_2_-damaged conditions, as shown in Figure 3B. When HEK293 cells were treated with 400 μM H_2_O_2_, cell viability significantly decreased, indicating that H_2_O_2_ can significantly reduce cell activity. However, after treatment with the same concentration of BSA@MnS, the cell viability of this group was not significantly different from that of the H_2_O_2_-damaged group. On the other hand, both CAT and CAT@MnS significantly increased cell viability, and CAT@MnS showed more significant improvement in cell viability.

Subsequently, we conducted cell cycle and apoptosis analysis using flow cytometry. The results showed that significant changes in the cell cycle occurred between the control and injury groups (Figures 3C,D). Following H_2_O_2_ treatment, the proportion of G0/G1 phase cells in HEK293 cells significantly decreased, while the proportions of S phase and G2/M phase cells markedly increased. Conversely, in the CAT@MnS pretreatment group, there was an increase in the proportion of G0/G1 phase cells and a decrease in the proportions of S phase and G2/M phase cells compared to Control group, confirming that CAT@MnS can alleviate cell cycle arrest at S phase and G2/M phases to protect against oxidative stress-induced damage. Simultaneously, the drug groups without H_2_O_2_ treatment further confirmed that neither CAT@MnS nor BSA@MnS exhibited significant cytotoxicity toward normal cells, nor did they cause cell cycle arrest (Supplementary Figures S6A,B).

To assess the impact of the treatments on cell proliferation, we performed flow cytometry analysis of Ki-67 expression (Supplementary Figures S6C,D). Compared to the Control group, H_2_O_2_ treatment significantly reduced the proportion of Ki-67-positive cells, indicating cell cycle arrest or proliferation inhibition. CAT@MnS significantly reversed this H_2_O_2_-induced proliferation suppression. In contrast, BSA@MnS and free CAT offered minimal protective effects. To further demonstrate the protective effect of CAT@MnS on apoptosis in HEK293 cells, we utilized flow cytometry for quantitative analysis of apoptosis in different treatment groups. As shown in Figure 3E, 93.9% of cells in the control group were viable, while the apoptosis rate was 48.6% in the H_2_O_2_-damaged group. The CAT@MnS and CAT pretreatment groups exhibited apoptosis rates of 7.25% and 17.59%, respectively.

To further verify the apoptosis rate quantified by flow cytometry, TUNEL staining was performed on HEK293 cells (Supplementary Figure S6E). Consistent with the flow cytometry analysis, TUNEL staining provided direct morphological evidence of apoptosis across the different groups. Compared to the control group, the H_2_O_2_-treated group showed a significant increase in TUNEL-positive cells, confirming extensive H_2_O_2_-induced apoptotic cell death. In contrast, the CAT@MnS group exhibited a remarkable reduction in TUNEL-positive cells compared to the H_2_O_2_ group, demonstrating its potent protective effect against apoptosis. On the other hand, the protective effects conferred by BSA@MnS and free CAT were less pronounced than that of CAT@MnS.

To confirm the ability of CAT@MnS to effectively eliminate intracellular ROS as anticipated, we conducted validation using an assay kit for reactive oxygen species (ROS). As depicted in Figure 3F, the H_2_O_2_-damaged group exhibited intense green fluorescence. However, in the CAT@MnS and CAT-pretreated groups at a drug concentration of 5 μg/mL, there was a noticeable decrease in intracellular green fluorescence levels, while the BSA@MnS pretreated group showed minimal change in fluorescence levels. These results indicate that CAT@MnS can significantly eliminate excessive ROS generated by H_2_O_2_ damage even at very low doses and demonstrate excellent clearance capability.

Furthermore, we assessed the changes in cell apoptosis following different drug treatments using a mitochondrial membrane potential assay kit (JC-1). As depicted in Figure 3G, HEK293 cells exposed to H_2_O_2_ exhibited a significant decrease in mitochondrial membrane potential. The green fluorescence intensity of the BSA@MnS group was comparable to that of the H_2_O_2_ group. Conversely, both the CAT@MnS and CAT pretreatment groups showed an increase in red fluorescence intensity and a decrease in green fluorescence intensity, resulting in fluorescence levels like those of the negative control group.

### 3.4 *In vivo* therapeutic efficacy of CAT@MnS on AKI mice

**FIGURE 4:**
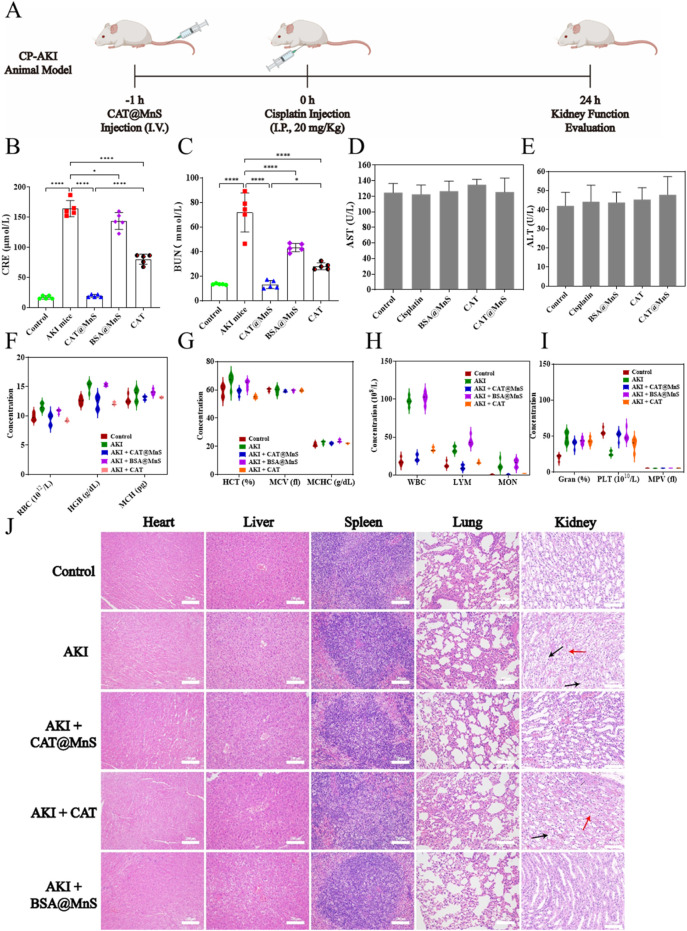
The preparation of cisplatin induced acute kidney injury mouse model and the correlation index of their organs and blood samples. **(A)** Schematic diagram of cisplatin induced acute kidney injury mouse model. **(B,C)** Kidney function detection of mouse in different treatment groups, n = 3. **(D,E)** Liver function detection of mouse in different treatment groups, n = 3. **(F–I)** Blood biochemical indexes detection of mouse in different treatment groups, n = 3. **(J)** H&E staining of major organs (heart, liver, spleen, lung, kidney) of mouse in different treatment groups. Representative images showing cellular vacuolization (black arrows) and disruption of the renal tubular basement membrane (red arrows). Scale bar, 100 μm.

Considering the satisfactory ROS-clearing ability and good biocompatibility of CAT@MnS, we proceeded to investigate its *in vivo* therapeutic effects on AKI (Figure 4A). To establish a mouse model of acute kidney injury (AKI), all female Balb/c mice were administered a single injection of 20 mg/kg cisplatin. The mice in each group were euthanized 24 h post-injection, and their organs and blood samples were collected for analysis.

Blood creatinine (CRE) and blood urea nitrogen (BUN), as nitrogenous end products mainly metabolized from the kidney, accumulate in the blood when the kidney tissue is damaged and the filtration ability is reduced, therefore, CRE and BUN are widely used in clinical kidney function detection, and are the gold standard for diagnosing acute kidney injury. As shown in Figures 4B,C, the levels of creatinine (CRE) and blood urea nitrogen (BUN) in the AKI group mice were significantly higher than those in the control group. The CRE and BUN levels in the CAT@MnS group were significantly lower than those in the AKI group, with no statistically significant difference compared to the control group. The BSA@MnS group and CAT group showed only a slight decrease in CRE and BUN levels compared to the control group, indicating that CAT@MnS has excellent therapeutic effects on AKI.

To further verify the biocompatibility and anti-inflammatory effect of CAT@MnS, we collected blood samples from mice in different drug treatment groups to detect the changes in blood biochemistry under different drug treatment conditions. As shown in Figures 4D–I, compared with the normal control group, there were no significant changes in blood biochemical indexes such as red blood cells, hemoglobin and platelets in the CAT@MnS group, which confirmed that CAT@MnS had good safety and biocompatibility. Evaluation of blood biochemical indicators revealed a sharp rise in white blood cell count in the AKI group (Figure 4H), suggesting a successful cisplatin-induced inflammatory response in mice. Comparing the white blood cell values of the control group and the CAT@MnS treatment group, the results showed that the white blood cell level of the CAT@MnS treatment group tended to be normal. The above experimental results demonstrate that CAT@MnS exhibits excellent biocompatibility and anti-inflammatory properties, making it a highly promising nanomedicine.

HE staining is the most intuitive experimental method to observe tissue and organ damage. To further evaluate whether CAT@MnS caused damage to normal tissues and organs and to judge the therapeutic effect of CAT@MnS on kidney organ damage, we performed H&E staining of heart, liver, spleen, lung and kidney of mice in different drug treatment groups. As shown in Figure 4J and Supplementary Figure S7A, there was no obvious pathological damage to the heart, liver, spleen, lung, brain and other organs in the CAT, CAT@MnS and BSA@MnS groups, proving that the CAT@MnS nanoclusters had no obvious toxic side effects in the organism. PAS and H&E staining revealed intact renal tubule structure with clear brush borders in the Control group (Figure 4J; Supplementary Figure S7B). In stark contrast, the AKI group displayed classic features of acute tubular injury, including widespread brush border loss, tubular dilation, and abundant cast formation, confirming successful model establishment. Treatment with CAT@MnS significantly ameliorated these pathological changes. The tubules exhibited markedly better structural integrity, a substantial reduction in cast formation, and improved preservation of the brush border, demonstrating the potent renoprotective effect of CAT@MnS. By comparison, BSA@MnS and free CAT exhibited only marginal efficacy. Collectively, these morphological findings provide compelling evidence that CAT@MnS nanoparticles effectively mitigate renal tubular injury in AKI.

**FIGURE 5:**
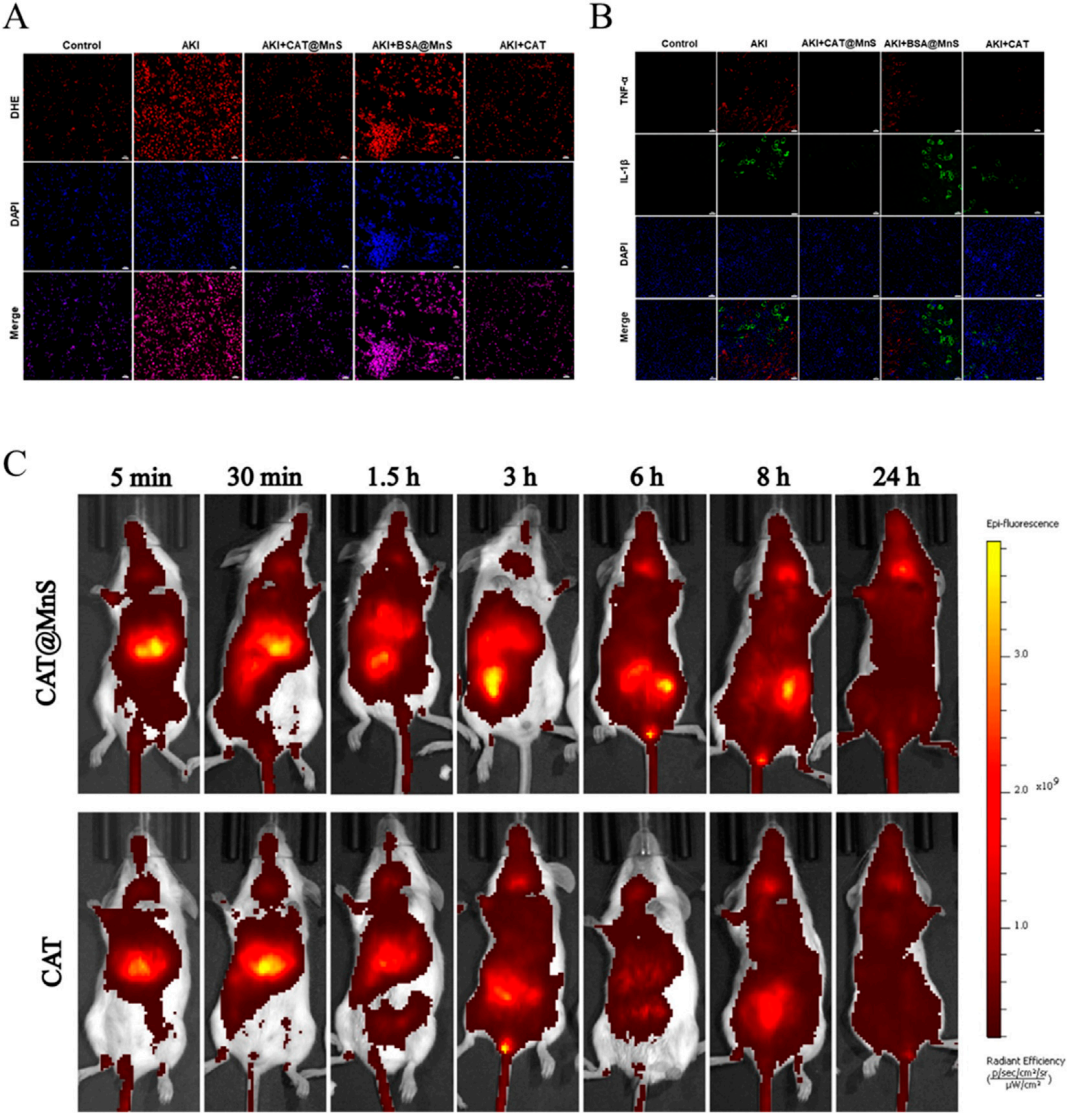
Fluorescence of mouse kidney and Drug distribution change *in vivo*. **(A)** Immunofluorescence images of ROS staining in kidney after treatment with different groups, Scale bar, 20 μm. n = 3. **(B)** CAT@MnS ameliorates cisplatin-induced inflammatory response in mouse kidneys. Representative immunofluorescence images of kidney sections from different treatment groups stained for the pro-inflammatory cytokines TNF-α (red) and IL-1β (green). Cell nuclei are counterstained with DAPI (blue). Scale bar, 100 μm. n = 3. **(C)** Time-dependent *in vivo* distribution of CAT@MnS and CAT. Representative fluorescence images of mice at the indicated time points post-injection. , n = 3.

To verify whether CAT@MnS could remove excess ROS produced by kidney tissue, we performed ROS fluorescence staining of kidney tissue from mice and imaged using laser confocal microscopy. The results showed that the level of red fluorescence in the kidneys of cisplatin-induced AKI mice was significantly higher than that in the negative control group. In addition, compared with the BSA@MnS treatment group and the AKI group, the CAT@MnS treatment group showed a significant reduction in ROS content in the kidney (Figure 5A; Supplementary Figure S8). This confirmed that CAT@MnS can protect mouse renal tubular cells by scavenging excessive ROS in the mouse kidney, and then treat AKI mice.

Tumor necrosis factor-α (TNF-α) and interleukin-1β (IL-1β) are important inflammatory mediators in organisms, which participate in the occurrence and development of inflammatory responses in organisms. IL-1β is mainly involved in local immune regulation, activating immune cells and causing the release of inflammatory mediators. TNF-α can promote inflammatory response, cell proliferation and differentiation, cell necrosis and apoptosis. Therefore, we verified the therapeutic effect of CAT@MnS in mice *in vivo* by fluorescent staining for TNF-α and IL-1β in kidney tissue and using laser confocal microscopy imaging. Immunofluorescence analysis indicated a marked upregulation of TNF-α and IL-1β in the kidneys of AKI mice, confirming the successful induction of renal inflammation by cisplatin. Compared with the AKI group, a significant reduction in fluorescence intensity was observed in the CAT@MnS-treated group, demonstrating the effective suppression of these pro-inflammatory cytokines. In contrast, the BSA@MnS control group failed to mitigate the inflammatory response, corroborating the potent anti-inflammatory effect of CAT@MnS in the kidneys of AKI mice (Figure 5B). To investigate the enrichment capability of CAT@MnS nanoparticles in renal tissue, we performed sequential imaging on experimental mice using an animal imaging system. We administered CAT@MnS and CAT into two groups of mice and monitored their distribution at various time points: 5 min, 30 min, 1.5 h, 3 h, 6 h, 8 h, and 24 h (Figure 5C). After intravenous injection in the tail vein, the enrichment of CAT@MnS nanoparticles in the kidneys was significantly higher than that of the natural CAT enzyme, with the most evident results observed at 3 h, 6 h, and 8 h. This indicates that the synthesized CAT@MnS nanoparticles can accumulate in the kidneys over a prolonged period and exert therapeutic effects even at low doses.

**FIGURE 6:**
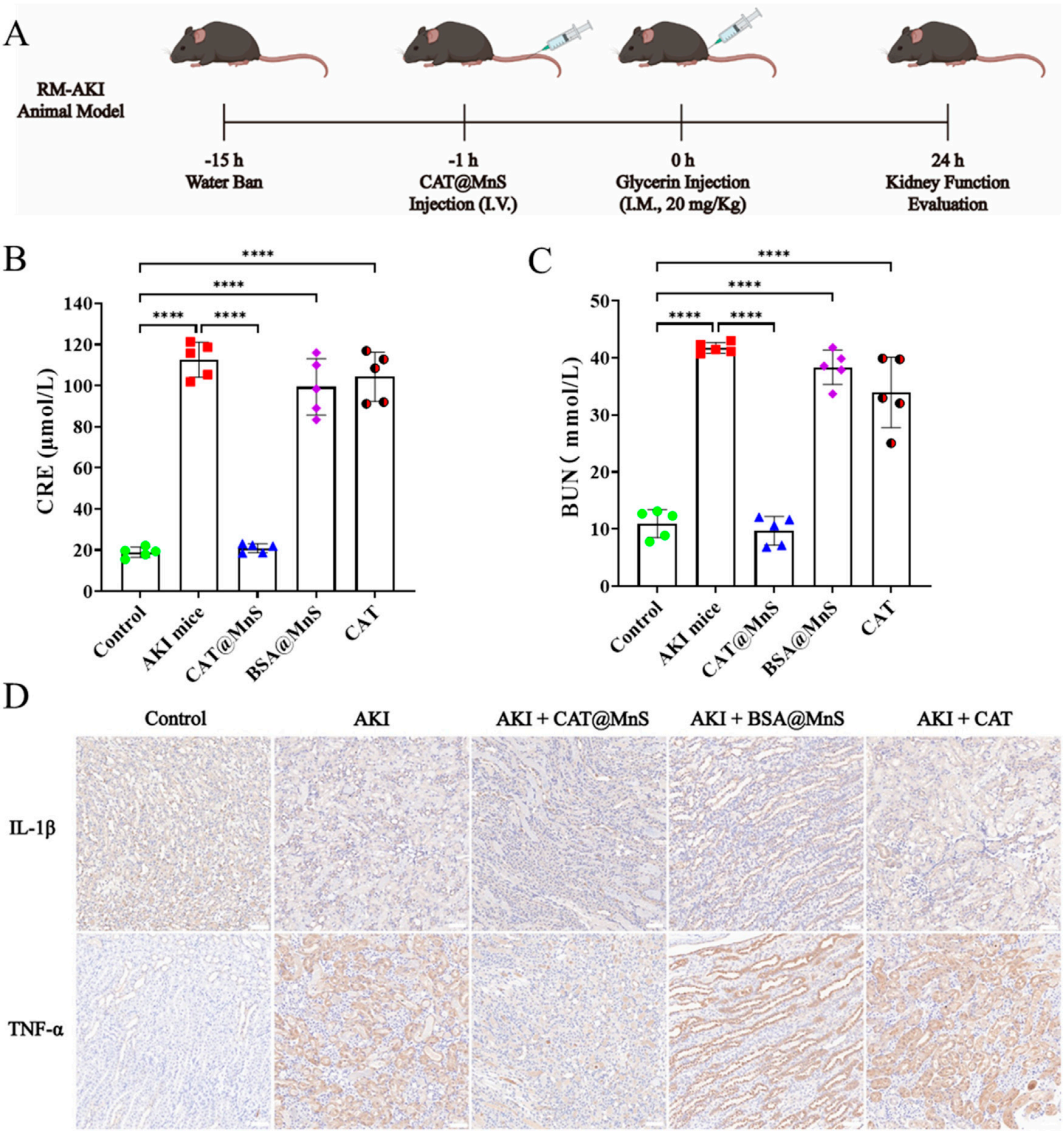
The preparation of glycerin induced acute kidney injury mouse model and the correlation index of their organs and blood samples. **(A)** Schematic diagram of glycerin induced acute kidney injury mouse model. **(B,C)** Kidney function detection of mouse in different treatment groups, n = 3. **(D)** Immunofluorescence images of TNF-α and IL-1β in kidney after treatment with different groups, n = 3.

Rhabdomyolysis syndrome refers to the destruction of striated muscle due to various reasons, myoglobin in the muscle is released in the blood, and subsequently circulates with the blood throughout the body. When myoglobin in the kidney is heavily filtered through the glomerulus to the proximal tubule of the kidney, it will cause oxidative stress and produce a large amount of ROS, which will subsequently damage the proximal tubular cells of the kidney and lead to AKI. Therefore, based on the encouraging results mentioned above, our subsequent research focus is to evaluate the effectiveness of CAT@MnS *in vivo* in a model of acute kidney injury induced by rhabdomyolysis (Figure 6A).

As shown in Figures 6B,C, the concentrations of CRE and BUN in AKI mice were higher than those in the Control group, and the levels of BUN and CRE in the CAT@MnS group were lower than those in the AKI group and were almost the same as those in the control group. Finally, to confirm the CAT@MnS can reduce the RM-AKI kidney tissues in the model expression of inflammatory factor, we have different drug treatment group mice kidney tissue of IL-1β and TNF-α testing two kinds of inflammatory factors, the results are shown in Figure 6D. The CAT@MnS drug treatment group was almost identical to the control group, proving that CAT@MnS did not cause a significant inflammatory response in the kidney. However, the levels of IL-1β and TNF-α in AKI mice were significantly higher than those in negative control group. In conclusion, CAT@MnS can reduce the expression of inflammatory factors such as TNF-α and IL-1β in kidney tissue, prevent diffuse fibrosis in kidney, and have strong anti-fibrosis and anti-inflammatory effects in RM-AKI mouse model.

In summary, we successfully fabricated a natural enzyme loaded CAT@MnS using a biomimetic mineralization approach and verified its role in the synergistic treatment of anti-oxidation and gas enhancement in AKI through a series of drug characterization and *in vitro* and *in vivo* experiments. Our results show that CAT@MnS has a small nanoparticle size, which can pass through the glomerular filtration barrier and accumulate in the kidney. At the same time, CAT@MnS can be stable at higher temperatures, extreme acid-base environments, and different ionic environments, thereby achieving a longer circulation time *in vivo* than the free CAT enzyme. And because CAT@MnS can release H_2_S gas slowly and continuously, H_2_S gas can be retained in the injured site for a long time. Further studies *in vitro* and *in vivo* have confirmed that CAT@MnS has strong antioxidant properties and can remove a large number of reactive oxygen species generated at the injured site, thereby protecting cells from oxidative stress damage. As a new type of antioxidant, this natural enzyme nanocomponent overcomes the limitations of traditional natural enzymes. By comparing the therapeutic effects of CAT@MnS and natural CAT enzymes, we clearly found that CAT@MnS has a stronger anti-inflammatory effect than the natural CAT enzyme. The H_2_S gas released by CAT@MnS can enhance the activity of CAT enzyme and play an anti-inflammatory role to achieve a synergistic antioxidant therapeutic effect. Overall, this work provides new guidance and new ideas for the search for biodynamic nanoplatforms to facilitate gas-synergistic antioxidant therapy for AKI and other inflammatory diseases.

## Data Availability

The original contributions presented in the study are included in the article/Supplementary Material, further inquiries can be directed to the corresponding author.
