# Supplementary material for: Nanozyme-armored natural enzymes for acute kidney injury management via inflammation regulation and oxidative damage mitigation
Source: Front Pharmacol. 2025 Dec 18;16:1686323. doi: 10.3389/fphar.2025.1686323 (PMC12756118; doi:10.3389/fphar.2025.1686323)
Supplement: Supplementary file 1 [file Supplementaryfile1.docx]

**Nanozyme-Armored Natural Enzymes for Acute Kidney Injury Management via Inflammation Regulation and Oxidative Damage Mitigation**

**Supporting information**

**Materials**

Bovine serum albumin (BSA), Catalase (CAT), Sodium sulfide (Na_2_S), Iron chloride hexahydrate (FeCl_3_·6H_2_O), Cupric chloride dihydrate (CuCl_2_·2H_2_O), Nickel chloride hexahydrate (NiCl_2_·6H_2_O), Zinc chloride (ZnCl_2_), Manganese Chloride Tetrahydrate (MnCl_2_·4H_2_O) were purchased from Shanghai Macklin Biochemical Technology Co., Ltd (China). Hydrogen peroxide (H_2_O_2_, 30%), ABTS, and DCFD-A were bought from Sigma-Aldrich (USA). Reactive Oxygen Species Assay Kit, Apoptosis Detection kit (propidium iodide/annexin V-FITC) were obtained from the Beyotime Institute of Biotechnology (China). All the aqueous solutions were prepared using purified deionized (DI) water purified with a purification system (Direct-Q3, Millipore, United States).

**Synthesis of BSA@MnS.**

Briefly, 10 mg bovine serum albumin (BSA), 7.3 mg manganese chloride (MnCl_2_), and 8.9 mg sodium sulfide (Na_2_S) were measured. MnCl_2_ and Na_2_S were dissolved in 1mL deionized water by magnetic stirring until completely dissolved and then placed for later use. BSA was dissolved in 1.2 mL deionized water and placed on a vortex oscillator to oscillate until the BSA solution was completely dissolved. Add 0.1 mL MnCl_2_ solution in shangbu solution, violent mixing after 15 min, slowly add 0.15 mL of Na2S solution; After the mixture solution was placed at 4℃ for 3 hours, the solution turned black. The black solution was dialyzed in deionized water for 24 hours using a dialysis bag with a truncated molecular weight of 8000-14000, and then cooled and dried in a lyophilizing machine to obtain BSA@MnS nanomaterials.

**Synthesis of CAT@MnS.**

Natural catalase (CAT) 10mg, manganese chloride (MnCl_2_) 7.3 mg, and sodium sulfide (Na_2_S) 8.9 mg extracted from bovine liver were weighed. MnCl_2_ and Na_2_S were dissolved in 1mL deionized water by magnetic stirring until completely dissolved and then placed for later use. The CAT enzyme was dissolved in 1.2 mL deionized water and shaken on a vortex oscillator until the CAT enzyme solution was completely dispersed. After centrifugation at 8000 rpm for 5 min, the supernatant was taken for further use. 0.1 mL MnCl_2_ solution was added to the supernatant of the previous step, and after vigorous stirring for 15 min, 0.15 mL Na_2_S solution was slowly added. After the mixture solution was placed at 4℃ for 3 hours, the solution turned black. After 24 hours of dialysis using a dialysis bag with a molecular weight cutoff of 8000-14000 in deionized water, the resulting material was dried in a lyophilizer to obtain CAT@MnS nanomaterials.

**Catalase-like Activity of CAT@MnS and BSA@MnS.**

The analyte were dissolved in deionized water to prepare 20 μg/mL solutions. The reaction was initiated by the sequential addition of 10 mL of PBS, 200 μL of the analyte, followed by 200 μL of 0.3% H₂O₂. Oxygen generation was monitored at 1-minute intervals over a period of 10 minutes. The oxygen generation curve was plotted with time as the X-axis and the oxygen generation (the difference in oxygen concentration at all time points from 0 min) as the Y-axis. The catalase activity was determined using a dissolved oxygen meter. The initial rate of oxygen production was recorded. One unit of catalase activity was defined as the amount of enzyme that produces 0.5 μmol of oxygen per minute under the assay conditions. The specific activity was expressed as units per milligram of protein (U/mg).

**ABTS radical-scavenging activity of CAT@MnS and BSA@MnS**

Mix the ABTS aqueous solution with potassium persulfate (K_2_S_2_O8) solution to prepare a mixed liquor concentration of 2.45 mM. Incubate in the dark at 4°C for 16 hours. The mixture is then combined with anhydrous ethanol. When the absorbance reaches 0.7±0.02, it becomes the ABTS working solution. Mix 10 μL of Nano-enzyme at different concentrations (0, 0.0625, 0.125, 0.25, 0.5, 1, 2, 4 mg/mL) with 190 μL of the ABTS working solution, incubate at room temperature for 6 minutes, and then measure the absorbance at 735 nm.

**Detection of H_2_S gas release**

First, a standard curve for the release of H_2_S gas was constructed by preparing solutions of different concentrations using an appropriate amount of Na_2_S crystals dissolved in deionized water. The content of H_2_S gas in these solutions was then measured using a hydrogen sulfide (H_2_S) content detection kit and a UV-visible spectrophotometer to determine the absorbance (OD value). The X-axis represented the H_2_S gas content, while the Y-axis represented the OD values measured by the UV-visible spectrophotometer. The CAT@MnS nanomaterial was dispersed in a buffer solution with a pH of 6.5 at a concentration of 1 mg/mL and gently agitated at 37°C. Subsequently, the H_2_S content in 2 mL of CAT@MnS solution was monitored at time intervals of 0.5, 1, 2, 3, 4, 5, 6, and 8 hours. The corresponding OD values were determined using the H_2_S content detection kit within the UV-visible spectrophotometer and were used to calculate the corresponding H_2_S gas content based on the previously established standard curve for H_2_S gas release.

**Hemolysis Assay of CAT@MnS and BSA@MnS**

Prepare Nano-enzyme into six solutions with concentrations of 50, 100, 200, 300, 400, and 600 μg/mL. Mix 900 μL of Nano-enzyme solutions at different concentrations with 100 μL of fresh red blood cell suspension, and incubate at 37°C for 30 minutes. Set up a positive control (low ionic water, which will cause complete hemolysis of red blood cells) and a negative control (PBS, in which red blood cells maintain their normal morphology). After the incubation, remove the unhemolysed red blood cells by centrifugation, and take the supernatant to measure its absorbance at 578 nm. By calculating the hemolysis rate, the hemolytic ability of the nanomaterial can be quantitatively evaluated.

**Cell culture**

Human embryonic kidney 293 (HEK293) cells were purchased from the American Type Culture Collection (ATCC) and cultured under 5% CO_2_ at 37 °C in Dulbecco’s Modified Eagle Medium (DMEM) supplemented with 1% penicillin/streptomycin and 10% fetal bovine serum (FBS).

**CCK-8 Cell Proliferation and Cytotoxicity Assay**

The CCK-8 assay was employed to evaluate the protective effects of BSA@MnS, CAT enzyme, and CAT@MnS on HEK293 cell proliferation under conditions of hydrogen peroxide (H_2_O_2_)-induced damage.

Three experimental groups were established: (1) BSA@MnS + H_2_O_2_; (2) CAT + H_2_O_2_; (3) CAT@MnS + H_2_O_2_. Following digestion and centrifugation, HEK293 cells were resuspended in a complete culture medium at a concentration of 8×10^4^ cells/mL and plated at 100 μL per well in a 96-well plate using a complete culture medium. After plating, the cells were incubated for 24 hours. The original drugs BSA@MnS, CAT enzyme, and CAT@MnS were diluted to seven concentration gradients of 5, 10, 20 and 40 μg/mL using a complete culture medium. Subsequently removing the original culture medium from each well was followed by sequential addition of drug solutions at each gradient with a volume of50μL. The plate was then placed in a cell culture incubator for 30 minutes after which 50 μL of 800 μM H_2_O_2_ solution diluted in complete culture medium was added to each well before further incubation for 12 hours. The drug was applied at final concentrations of 2.5, 5, 10, and 20 μg/mL, while the final concentration of H₂O₂ was maintained at 400 μM. CCK-8 reagent was added post-incubation and OD values were measured using a Microplate reader.

**AKI mouse model**

The Balb/c female mice (6-8 weeks, 17-21g) used in this study were purchased from the Experimental Animal Center of Zhengzhou University, and all animal studies were conducted by the protocol approved by the Animal Ethics Committee of the Experimental Animal Center of Zhengzhou University (No. ZZU-LAC20220729[04]).

Glycerol-induced AKI mouse model: Balb/c mice were dehydrated for 15 hours but were able to freely obtain food. Afterward, 50% glycerol was injected into the muscles of both hind limbs of the mice at a dose of 8 mL/kg. After 2 hours of injection, an AKI mouse model was successfully established and used for subsequent experiments.

Cisplatin-Induced Acute Kidney Injury Model: cisplatin (20 mg/kg) injected into the abdominal cavity of all Balb/c mice. For the treatment group, mice were injected with BSA@MnS, CAT, and CAT@MnS through the tail vein 2 hours after intraperitoneal injection of cisplatin. After 24 hours, each group of mice was euthanized, and blood samples and kidney tissue were collected and analyzed.

**Therapeutic Effect in AKI Mice.**

To analyze the therapeutic effects of BSA@MnS, CAT, and CAT@MnS, we randomly divided mice into five groups: (i) healthy mice treated with PBS; (ii) AKI mice treated with PBS; iii) AKI mice treated with CAT; (iv) AKI mice treated with BSA@MnS; (v) AKI mice treated with CAT@MnS. After treatment, kidney function and body weight were monitored.

**Kidney Function Evaluation.**

After 24 hours of intravenous injection of BSA@MnS, CAT, and CAT@MnS, the mice were euthanized, and kidney and blood samples were collected. The obtained kidney sections were stained with H&E, and the levels of BUN and CRE in blood samples were detected.

**Evaluation of ROS Clearance Capacity**

To further assess the antioxidant capacity of CAT@MnS at a microscale level, we investigated the mechanism of action by examining the clearance of ROS in the kidneys. Initially, the kidneys from each experimental group of mice were extracted and stored at -80°C. Subsequently, cryosectioning was performed at 20°C to obtain kidney tissue sections with a thickness of approximately 5 μm. The frozen sections were washed with PBS and stained with DHE fluorescent probe for 30 minutes to detect superoxide anion formation.  Following this, coverslips were mounted on each slide using Vectashield Mounting Medium (Vector Laboratories, Burlingame, CA, USA), and confocal imaging was conducted using a laser scanning confocal microscope.

**Study of drug distribution in the body**

The normal mice were randomly divided into two groups (n=3): (1) CAT; and (2) CAT@MnS. Cy5.5-labeled natural CAT and CAT@MnS were injected via the tail vein at a drug-to-body weight ratio in both groups. The mice in group 1 received injections of natural CAT, while those in group 2 received injections of CAT@MnS. Live imaging was performed on the experimental mice at 5 minutes, 0.5 hours, 1 hour, 3 hours, 6 hours, 8 hours, and 24 hours post-administration using an in vivo imaging system.


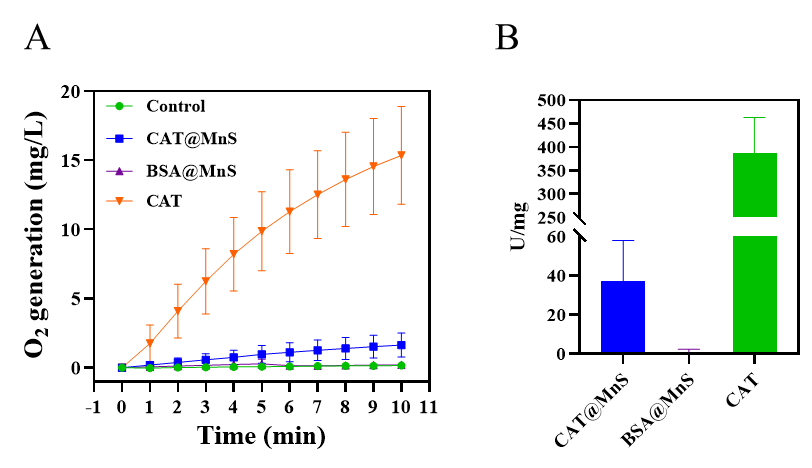


Figure S1:The catalase activity of CAT@MnS, BSA@MnS and CAT. A) Oxygen curves of CAT@MnS, BSA@MnS and CAT. B) Enzyme activity of CAT@MnS, BSA@MnS and CAT.

Figure S2: Antioxidative activities of CAT@MnS.Comparison of ABTS radical-scavenging rates of CAT@MnS and BSA@MnS materials at different concentrations (0.0625 - 4 mg/mL). Error bars represent standard deviations.


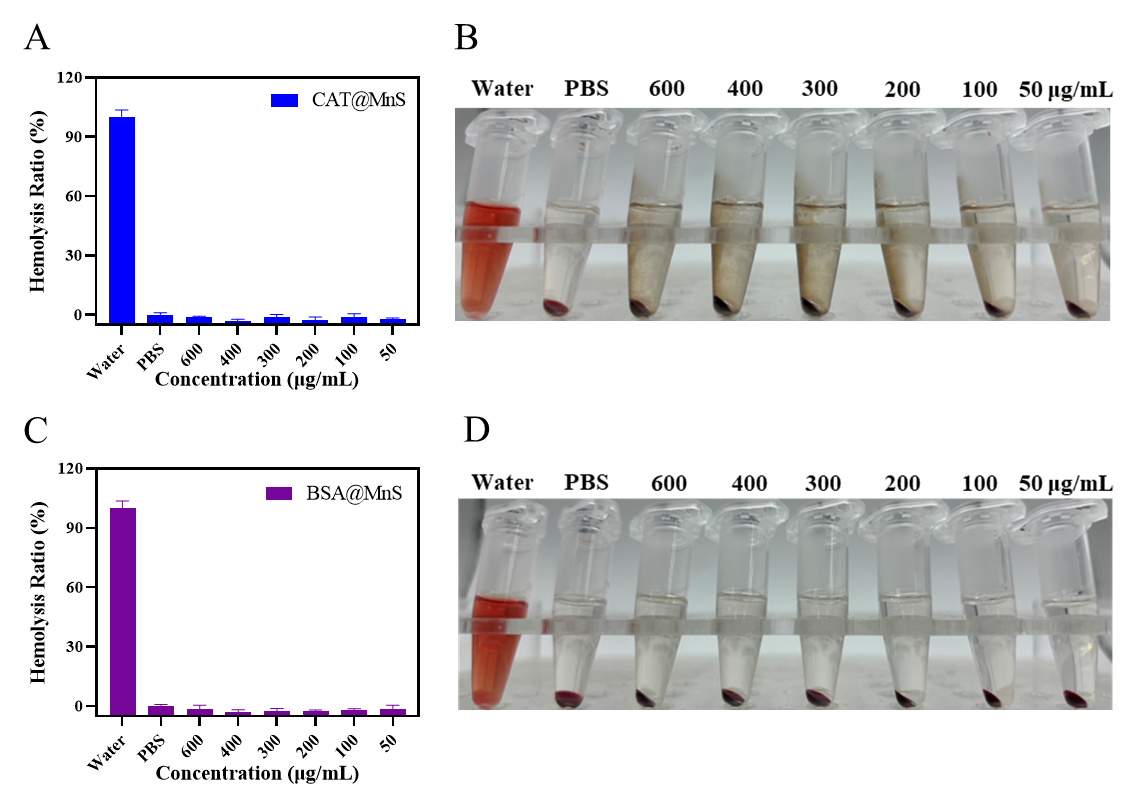


Figure S3: Hemolysis test results of CAT@MnS and BSA@MnS at various concentrations. A) Bar chart of hemolysis rates for CAT@MnS in deionized water, PBS buffer, and at various concentrations (600, 400, 300, 200, 100, and 50 μg/mL); B) Photographs of the corresponding conditions in A. C) Bar chart of hemolysis rates for BSA@MnS in deionized water, PBS buffer, and at various concentrations (600, 400, 300, 200, 100, and 50 μg/mL); D) Photographs of the corresponding conditions in C. Deionized water served as a positive control, while PBS buffer served as a negative control. The results indicate that within the tested concentration range, both materials exhibited low hemolysis rates under conditions other than deionized water.


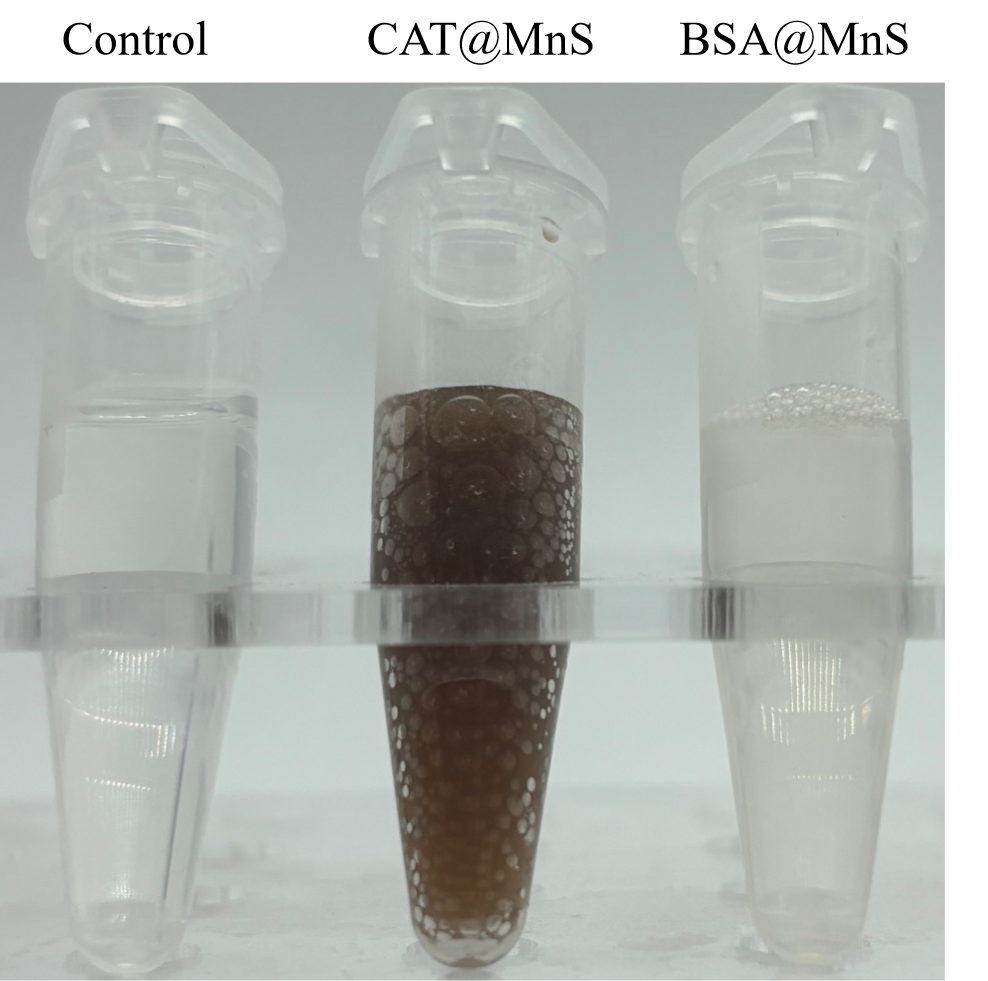


Figure S4: Oxygen generation experiments of CAT@MnS and BSA@MnS. The material concentration is 1 mg/mL, and the concentration of H₂O₂ is 4%.

Figure S5: Quantification of the relative fluorescence intensity shown in Figure 5A**,** n=7; Data are presented as means ± SD, ****p<0.0001


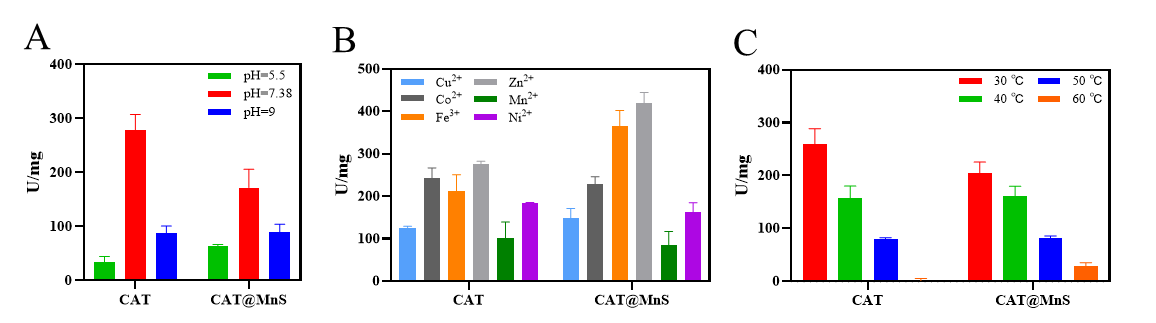


Figure S6: Catalase-like activity of CAT and CAT@MnS under different pH, metalions, and temperatures. A) Catalase-like activity of CAT and CAT@MnS under different ,n=3. B) Catalase-like activity of CAT and CAT@MnS under different metalions, n=3. C) Catalase-like activity of CAT and CAT@MnS under different temperatures, n=3.


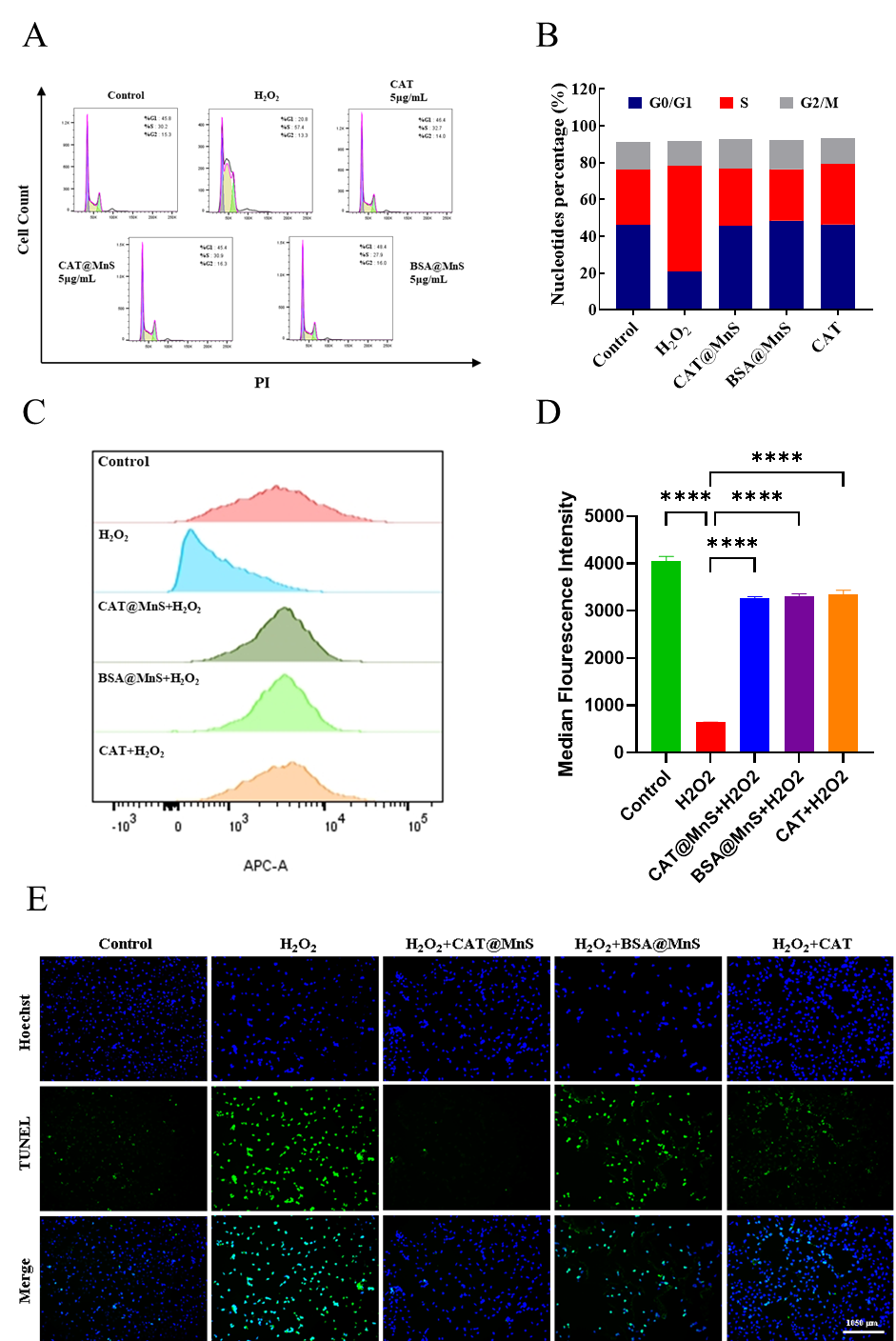


Figure S7: Antioxidant effects of CAT@MnS. A) and B) Cell cycle and apoptosis analysis after treatment with different groups, n=3. C) Representative histograms of Ki67 expression in each group under 400 μM H2O2 treatment. D) Quantitative analysis of the data shown in panel C. Data are presented as means ± SD, n=3, ****p<0.0001. E) Representative fluorescence images of TUNEL staining post-treatment in each group, n=3. Scale bar, 1050 μm.


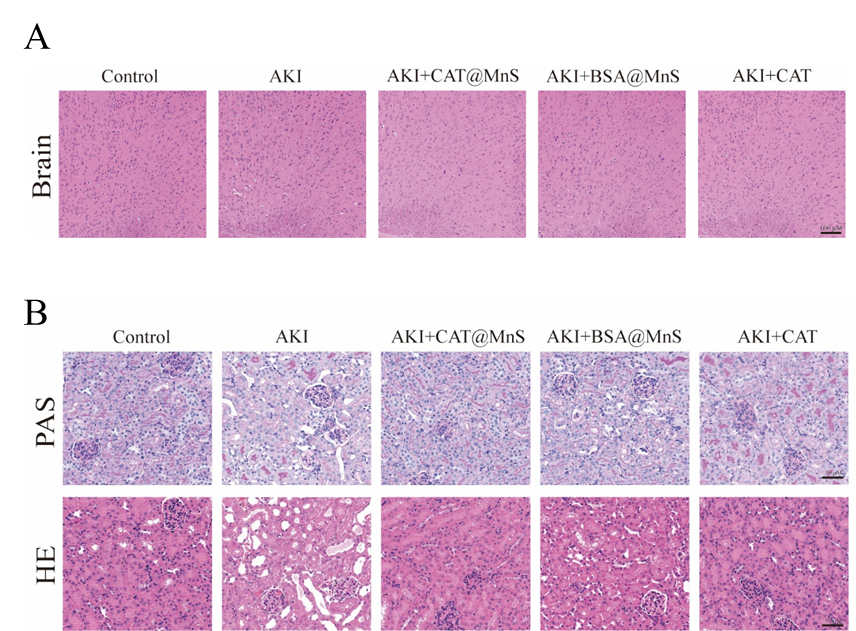


Figure S8: The preparation of cisplatin induced acute kidney injury mouse model and the correlation index of their organs and blood samples. A) Representative H&E staining of mouse brain sections across treatment groups. Scale bar, 100 μm. B) Representative PAS and H&E staining of renal tissues across treatment groups. Scale bar, 50 μm.

Table S1: The specific grouping of Figure S4.

|  | Control | CAT@MnS | BSA@MnS |
| --- | --- | --- | --- |
| DI water (mL) | 1 |  |  |
| CAT@MnS (mL) |  | 1 |  |
| BSA@MnS (mL) |  |  | 1 |
| H_2_O_2_ (μL) | 100 | 100 | 100 |
